# Choice of injection time of conscious sedation and its impact on pain control in colonoscopy

**DOI:** 10.3389/fsurg.2022.886129

**Published:** 2022-10-18

**Authors:** Mingli Su, Tingting Chen, Qinghua Zhong, Dezheng Lin, Wei Liu, Yuping Su, Jiaxin Deng, Jiawei Zhang, Jiancong Hu, Xuefeng Guo

**Affiliations:** ^1^ Department of Endoscopic Surgery, The Sixth Affiliated Hospital, Sun Yat-sen University, Guangzhou, China; ^2^ Guangdong Provincial Key Laboratory of Colorectal and Pelvic Floor Diseases, The Sixth Affiliated Hospital, Sun Yat-sen University, Guangzhou, China

**Keywords:** colonoscopy, conscious sedation and analgesia, injection time, visual analogue scale, pain

## Abstract

**Purpose:**

The aim of this study was to identify the effect of different injection times on pain during colonoscopy procedure.

**Methods:**

In this retrospective study, the data of patients who underwent colonoscopy from June 2020 to September 2020 were assessed to investigate the effect of different injection time of sedative drugs (midazolam and dezocine). The primary endpoint was evaluating the pain intensity of the patients using visual analogue scale (VAS) immediately after colonoscopy .

**Results:**

A total of 152 patients were eligible for this study. Of them, 76 received midazolam and dezocine injection 1 min prior to the colonoscopy procedure (the 1 Min group) and the other 76 patients received the injection 3 min prior to the procedure (the 3 Min group). The vital signs of all patients were stable except for one patient who was diagnosed with inflammatory bowel disease in the 3 Min group. A transient drop in blood pressure for this patient was observed during colonoscopy but returned to normal after general treatment. The two groups had similar rates of cecal intubation (84.21% vs. 90.97%, *P* = 0.22), addition of sedative drugs during procedure (2.63% vs. 5.26%, *P* = 0.68), and adequate bowel preparation (Boston Bowel Preparation Scale ≥6, 61.84% vs. 61.84%, *P* = 1.0). However, patients in the 3 Min group had significantly lower VAS than those in the 1 Min group [0 (0, 1) vs. 1 (0, 2), *P* = 0.041].

**Conclusion:**

The timing of drug injection during conscious sedation may affect pain control during colonoscopy, with 3 min prior to the procedure showing lower VAS.

## Introduction

Colorectal cancer (CRC) is a global public health issue, with an estimation of 1.93 million new cases and 930,000 deaths worldwide in 2020 (1, 2). Detecting colorectal lesion by fecal immunochemical test (FIT) or colonoscopy has been shown to effectively decrease the incidence of CRC (3). FIT is convenient and economical, while colonoscopy has a greater CRC incidence reduction (4) and is currently regarded as the gold standard examination for colorectal lesion screening (5, 6). The primary goals of these screenings are to identify and aptly remove colorectal adenomas and lesions that are believed to lead to the development of CRC (7, 8).

Colonoscopy often presents as a painful and unpleasant procedure, which is one of its major barriers for willingness among the public (9). Among procedure-related pain factors, sedation could greatly reduce pain and discomfort of the examinee during colonoscopy. Recommendations for sedation methods in routine colonoscopy differ across countries and regions (10). Two methods of sedation are commonly used for colonoscopy test: one is conscious sedation with midazolam, and the other is modified anesthesia with propofol (11–15). The latter method could induce a deeper level of sedation with less patient movement and awareness. However, deep sedation colonoscopy is associated with higher cost and greater risk of sedation- and procedure-related complications (16). Furthermore, modified anesthesia can only be performed by an anesthesiologist, which is one of the main reasons for the lower sedation rate in China, compared to the United States and Europe (17). Different from propofol, conscious sedation with midazolam can be managed by the endoscopist and has similar sedative effect to propofol (15). However, a few studies have investigated the safety and effectiveness of conscious sedation. Fox example, the relationship between injection time and the efficiency of conscious sedation and analgesia remains unclear.

In this study, we evaluated the safety of sedation with midazolam and dezocine and compared the efficiency of different injection times (1 or 3 min before colonoscopy procedure) using the visual analogue scale (VAS) immediately after colonoscopy.

## Materials and methods

### Patients and data collection

Patients older than 18 years who had undergone colonoscopy were eligible for inclusion, and patients with stage IV colorectal cancer, history of chronic pain, or who had incomplete or missing follow-up data were excluded. The patients were divided into two groups: group 1 (injection of midazolam and dezocine 1 min prior to colonoscopy) and group 2 (injection of midazolam and dezocine 3 min prior to colonoscopy). We retrospectively collected the clinical data of 200 people who underwent colonoscopy between June 2020 and September 2020 at the Six Affiliated Hospital of Sun Yat-sen University (Guangzhou, China). Propensity score matching was used to control selection bias and 152 patients were finally included in this study. The propensity score of being allocated to group 1 and group 2 was calculated using a multivariable logistic regression model with age and sex as covariates. The patients were matched using the nearest-neighbor method in a 1:1 ratio with a caliper width of 0.2 SD of the logit of the propensity score.

Demographic data including age, gender, cardiovascular disease history, diagnosis, history of colonoscopy screening, history of previous abdominal surgery, Boston Bowel Preparation Scale (BBPS), addition of sedative drugs during procedure, cecal intubation rate (CIR), resection of polyp, VAS, change in vital signs [blood pressure, pulse, and peripheral blood oxygen saturation (SPO_2_)], satisfaction of colonoscopy, and postoperative complications were collected.

### Colonoscopy procedure and conscious sedation and analgesia

Patients who were inpatients and outpatients received colonoscopy according to their diagnosis and clinical need. All patients received regular bowel preparation instructions at their appointment to ensure proper preparation for colonoscopy. Colonoscopy was performed by several experienced endoscopists. All patients were assessed to tolerate conscious sedation and analgesia and were divided into group 1 or group 2. Midazolam (0.05 mg/kg) and dezocine (5.0 mg) were intravenously injected by nurses from the endoscopic department 1 or 3 min prior to the beginning of colonoscopy. Vital signs including pulse, blood pressure, and blood oxygen saturation were recorded every 5 min during the procedure.

### Definition of the BBPS

To evaluate the quality of bowel preparation, the BPPS criteria was used. It ranges from a score of 0–9 and divides the colon into three regions (including right colon, transverse colon, and left colon) whereby each region receives a score from 0 to 3 respectively (18). An adequate bowel preparation is usually defined as a BBPS ≥6.

### Definition of VAS

VAS was used to measure the pain intensity of patients during the colonoscopy procedure (19). Patients would be transferred from the examination room to the resuscitation room after finishing colonoscopy procedure and then were asked to place a line perpendicular to the VAS line to present their pain intensity. The score was determined by measuring the distance between the “no pain” anchor and the patient's mark, ranging from 0 to 10.

### Statistical analysis

IBM SPSS (version 25.0 for Windows; SPSS, Chicago, IL, United States) was used for data analysis. Measurement data are expressed as the mean ± SD or median (minimum, maximum). The Mann–Whitney rank sum tests or *t*-tests were used to compare the measurement data between groups. The differences between rates were tested by the Chi-square test or Fisher exact tests, where appropriate. Differences were considered statistically significant for *P* values <0.05.

## Results

In this study, a total of 152 patients who underwent colonoscopy with conscious sedation between June 2020 and September 2020 were finally included after propensity score matching. Their basic characteristics are shown in Table 1. Of them, 76 patients received an injection of midazolam and dezocine 1 min prior to colonoscopy (defined as group 1) and the other 76 patients were 3 min prior to the procedure (defined as group 2). In group 1 and group 2, 48 and 39 male patients were included, respectively. The differences between the two groups are presented in Table 1. The two groups had similar characteristics of gender, age, cardiovascular disease history, history of colonoscopy procedure, and diagnosis. Benign perianal disease and colorectal polyp were the two most diagnosed in both groups.

**Table 1:** Comparison of the demographic and clinical characteristics of two groups.

Variables	Group 1 (*n* = 76)	Group 2 (*n* = 76)	*P*
Age, median	44.5	45.0	0.94
Male, *n* (%)	48 (63.15)	39 (51.32)	0.14
Diabetes	2 (2.63)	0 (0)	0.50
Cardiovascular disease, *n* (%)	3 (3.95)	4 (5.26)	1.0
Colonoscopy history, *n* (%)	50 (65.79)	40 (52.63)	0.099
Diagnosis, *n* (%)			
Benign perianal disease	27 (35.53)	27 (35.53)	
Colorectal polyp	20 (26.32)	15 (19.74)	
Physical examination	12 (15.79)	14 (18.42)	
Colorectal carcinoma	10 (13.16)	14 (18.42)	
Inflammatory bowel disease	7 (9.21)	6 (7.89)	

Table 2 illustrates the comparison of clinical outcomes of patients between the two groups. Patients in both groups had high satisfaction with the procedure. The two groups had similar rates of cecal intubation (84.21% vs. 90.97%, *P* = 0.22), addition of sedative drugs during the procedure (2.63% vs. 5.26%, *P* = 0.68), and adequate bowel preparation (BBPS ≥6, 61.84% vs. 61.84%, *P* = 1.0). However, patients in group 2 had a significantly lower VAS than those in group 1 [0 (0, 1) vs. 1 (0, 2), *P* = 0.041].

**Table 2:** Comparison of the clinical outcome of two groups.

Variables	Group 1 (*n* = 76)	Group 2 (*n* = 76)	*P*
BBPS ≥6	47 (61.84)	47 (61.84)	1.0
Cecal intubation rate	64 (84.21)	69 (90.79)	0.22
Length of colonoscopy (min), median (IQR)	22.0 (16.75, 29.25)	20.0 (14.75, 25.00)	0.075
Addition of drugs	2 (2.63)	4 (5.26)	0.68
Resection of polyp	30 (39.47)	26 (34.21)	0.50
VAS, median (IQR)	1 (0, 2)	0 (0, 1)	0.041
Satisfaction with colonoscopy	65 (85.52)	66 (86.84)	0.81

BBPS, Boston Bowel Preparation Scale; IQR, interquartile range.

**Figure 1:**
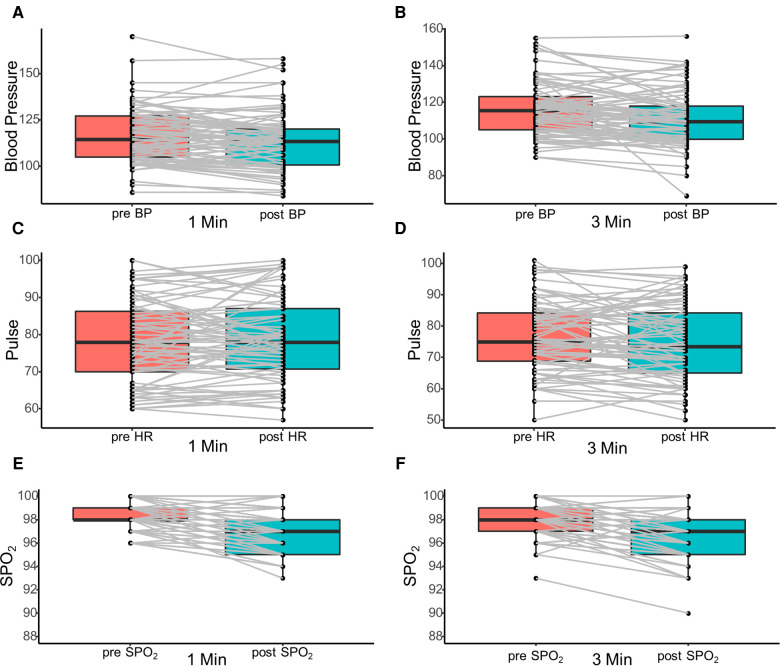
Illustration of the changes in arterial blood pressure (**A,B**), pulse (**C,D**), and SPO_2_ (**E,F**) before and after colonoscopy procedure. SPO_2_, peripheral blood oxygen saturation.

To further investigate the safety of the procedure, we have illustrated the changes in arterial blood pressure, pulse, and SPO_2_ in Figure 1. The vital signs of all patients remained stable during the procedure except for one patient in group 2 who had inflammatory bowel disease (IBD). There was a transient drop in blood pressure for this patient during the procedure and it quickly returned to normal after general treatment without interrupting the endoscopic procedure. Moreover, no patient had any postoperative complications such as intestinal bleeding, intestinal perforation, and bowel obstruction.

## Discussion

In this study, we found that conscious sedation and analgesia was safe and had a satisfactory level of comfort during the procedure, with a high satisfaction score (approximately 86.84%) and low rate of need for additional pain medication. In addition, intravenous injection of sedative drugs 3 min before the procedure had significantly better pain control than 1 min (*P* = 0.041). Patients’ pain and fear of colonoscopy are among the reasons for reluctance to colonoscopy (17). Main sedation during colonoscopy includes propofol sedation and conscious sedation with midazolam, which could decrease procedure-related pain and discomfort (15). Furthermore, clear and satisfying visualization under colonoscopy due to the patient's cooperation has led to increased satisfaction. The overall sedation rate for colonoscopy is only 47.9% in China, which is much lower than in the United States (>98.0%), Greece (78.0%), and Germany (91.0%) (17). Previous studies have reported that propofol sedation has some disadvantages, including severe respiratory depression and hypotension (10, 22). Conscious sedation with midazolam is efficient and safe and has been used widely during colonoscopy (21). Cancer burden has major economic implications both on the patient-level and country-level (22). Here, we found that compared with propofol, sedation administration by a nurse under endoscopist supervision during conscious sedation could help lower the costs of examination to the patient and might also lead to greater willingness for this procedure (10). However, the timing of drug injection for better pain control remains unclear. Thus, in this study, the 1 and 3 min injection times of sedation prior to colonoscopy were investigated. The results suggest that 3 min injection prior to the procedure had significantly lower VAS than 1 min (*P* = 0.041). The onset time of a sedative analgesic such as midazolam is relatively slower than propofol. This may be the reason why 3 min injection administration had better pain control effect than 1 min. Further studies are needed to see if lower VAS vary based on time and level of sedation administered.

Several factors including sedation, adequate bowel preparation (based on BBPS ≥6), previous abdominal surgery, and endoscopists’ experience were identified in previous studies to be associated with pain during procedure (23, 24). In this study, the procedural technique was the same for each endoscopist in both the 1 and 3 min groups at the same academic teaching institution. The rate of adequate bowel preparation (BBPS ≥6) for all patients was 60.9%, which did not have lower VAS than the patients with BBPS <6 (*P* = 0.76).

Similarly, the quality of bowel preparation, the use of sedation, and the endoscopist's experience were the main factors influencing CIR (25). Conscious sedation provides the endoscopists time to focus on the examination and not be distracted by a patient’s incorporation. No significant differences were noted considering the CIR between two groups in this study (*P* = 0.22). However, patients with adequate bowel preparation (BBPS ≥6) had a higher rate of reaching the cecum than other patients (*P* = 0.001). An adequate bowel preparation could decrease the procedure time and make endoscope insertion easier, thereby having a higher rate of cecal intubation and better procedure tolerance (26)*.* Furthermore, adequate bowel preparation may facilitate complete gas removal during colonoscope withdrawal, thereby contributing to pain control during the procedure.

Except for pain during the procedure, several factors such as previous experience with colonoscopy and appropriate level of sleep were also associated with satisfaction of the procedure (13). In this study, 59.2% patients had previous experience with colonoscopy, which had a similar satisfaction of colonoscopy comparing with those who received colonoscopy for the first time (*P* = 0.516). In addition, patients who underwent polyp resection during colonoscopy had similar median VAS scale with others. It was reported that patients with inflammatory bowel disease favored propofol sedation over conscious sedation (16). Moreover, IBD patients (*n* = 15) presented similar outcomes compared to non-IBD patients (*n* = 137) in our study (*P* = 0.75).

The limitation of this study was its retrospective nature and single institution data with relatively small sample size. Large prospective large-sample-size and multicenter studies are required to further evaluate the timing of drug injection and outcomes.

## Conclusion

The timing of drug injection during conscious sedation may affect the pain control during colonoscopy, with 3 min prior to the procedure showing lower VAS.

## Data Availability

The raw data supporting the conclusions of this article will be made available by the authors, without undue reservation.
